# Stent dilatation of atretic aortic coarctation in an adult-case report and literature review

**DOI:** 10.1186/s13019-016-0395-1

**Published:** 2016-01-19

**Authors:** Neerod Kumar Jha, Magdi Tofeig, Rajappan Arun Kumar, Amin ElTahir, Syed Mohammad Athar, Aref AlHakami, Arshad Khan, Mohammad Daud Khan

**Affiliations:** Institute of Cardiac Sciences, Sheikh Khalifa Medical City (managed by Cleveland Clinic), PO Box 51900, Abu Dhabi, United Arab Emirates; Institute of Anesthesiology, Sheikh Khalifa Medical City (managed by Cleveland Clinic), PO Box 51900, Abu Dhabi, United Arab Emirates; Institute of Surgery, Sheikh Khalifa Medical City (managed by Cleveland Clinic), PO Box 51900, Abu Dhabi, United Arab Emirates; Institute of Medicine, Sheikh Khalifa Medical City (managed by Cleveland Clinic), PO Box 51900, Abu Dhabi, United Arab Emirates

**Keywords:** Aorta, Coarctation, Stent, Dilatation, Cardiac

## Abstract

**Background:**

Patients with functional aortic interruption of the descending thoracic aorta at the isthmus due to severe coarctation in association with atretic lumen are extremely rare in the adult population. The management is challenging and carries high morbidity and mortality.

**Case presentation:**

We describe successful percutaneous reconstruction using a covered stent in a similar patient who is doing well two-years after intervention. A literature search was done to explore management strategies and their long-term outcomes for better understanding.

**Conclusions:**

This report is an attempt to highlight the role of minimal invasive approach in the management of rare, severe coarctation of the aorta in adult patients to avoid morbidity and mortality associated with more invasive procedures.

## Background

Patients with functional aortic interruption of the descending thoracic aorta at the isthmus due to severe coarctation of aorta (CoA) and atresia are extremely rare and seldom attain advance age [1–4]. Coarctation of the aorta accounts for 5 to 10 % of all congenital cardiovascular malformations [1]. The available treatment modalities include surgery, balloon angioplasty and endovascular stenting [1–13]. However, management strategies are debatable due to limited and variable long-term outcomes [10–13]. We report herewith, an adult male with severe CoA of proximal descending aortic segment associated with atretic lumen, which underwent successful percutaneous reconstruction using a covered stent and required re-intervention later.

## Case presentation

A 27-year-old male with recently diagnosed severe CoA was referred to us with history of refractory hypertension and shortness of breath (NYHA II) of three years duration. There was no history of trauma or surgery. Also, there was no claudication. On physical examination, the blood pressure in the right upper limb was 200/120 mm Hg. A radio-femoral delay was apparent and a pressure gradient of 90 mm Hg was noted between the upper and lower extremities. The pulsations in the lower limbs were faint. Routine blood chemistry and urine analysis was un-remarkable. A chest x-ray delineated rib notching. A 2-D echocardiogram revealed a bicuspid aortic valve with mild stenosis and left ventricular hypertrophy with mild impairment. A contrast CT angiogram showed a severe CoA of the descending aorta at the isthmus involving a 4 cm long segment with merely 1–2 mm lumen and a gradient of 90 mm Hg with a diastolic runoff.

**Fig. 1:**
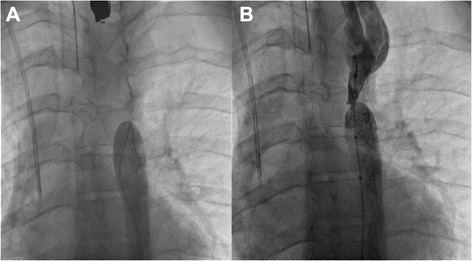
**a** Descending aortogram showing a blind distal aortic segment below the involved segment. **b** Descending aortogram showing a guide-wire across the atretic membrane of the aortic lumen

A decision was made to proceed with a transcatheter stent implantation at the Coarctation/atretic aortic segment under general anaesthesia. A 14 F sheath was placed in the right femoral artery via a surgical cut down. An initial angiogram revealed a blind end at the level of the coarctation (Fig. 1a).

The initial management plan was to approach via right axillary artery with a “kissing technique” to perforate the atretic segment. However, while manipulating a straight Terumo™ wire through the distal part of the aorta, incidentally the wire went through the membranous atretic lumen of the aorta. The diameter of atretic aortic lumen was 1.2 mm (Fig. 1b).The subsequent angiogram however confirmed the absence of any resultant vascular complication. Therefore, we proceeded further.

**Fig. 2:**
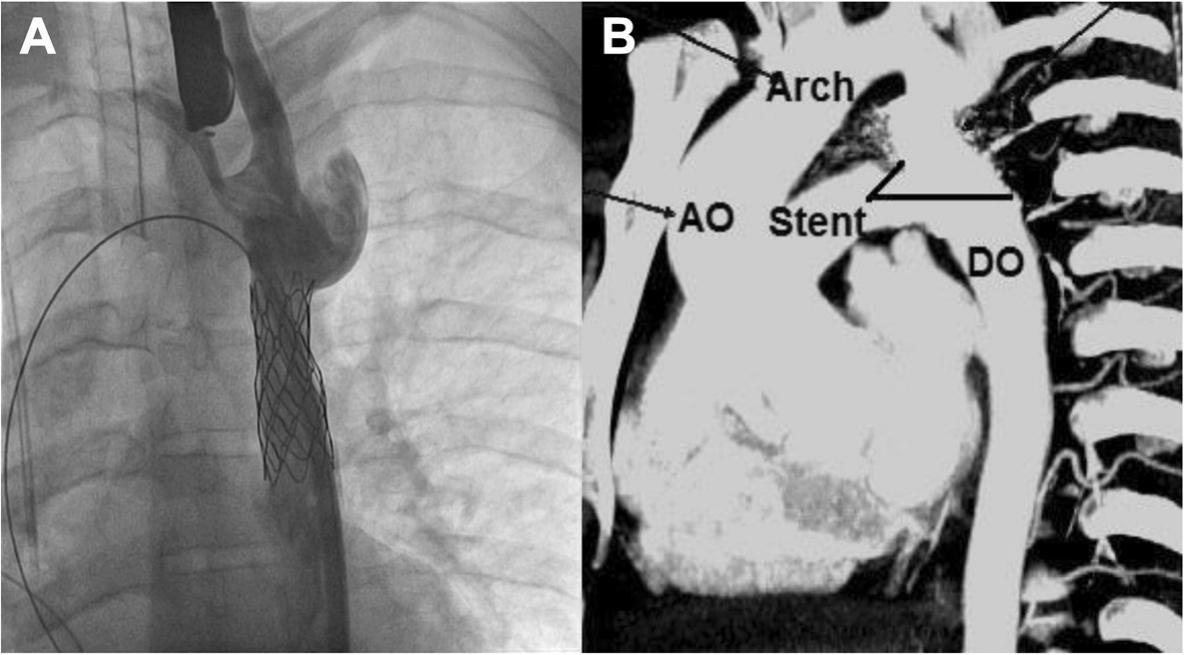
**a** Aortogram showing placement of a stent across the coarctation of the aorta. **b** Follow-up CT angiogram showing a patent stent and the lumen of the aorta. (AO- Ascending aorta, DO- descending aorta)

A 45 mm PTFE covered Cheatham-Platinum stent was positioned successfully at the coarctation through a 14 F Mullins sheath. A 16 mm X 4 cm Z-Med™ balloon was used to successfully inflate the stent distal to the left subclavian origin (Fig. 2a). Post implantation gradient was noted to be about 3 mm Hg. The patient went on to make an uneventful recovery and was discharged home. At 1-year follow up, an aortogram revealed good flow in the involved segment without any significant gradient. At 2-year follow up, an echocardiographic evidence of high gradient across the aortic valve (60 mm Hg) and progressive dyspnea on exertion prompted us to perform a cardiac catheterization. A narrowing of the stent was found at the middle segment with a measured pull back gradient of 10 mm Hg across it. We proceeded to re-dilate the stent successfully (Fig. 2b). The aortic valve gradient was 30 mm Hg. Currently, the patient is asymptomatic and receiving anti-hypertensive medications in addition to aspirin and ACE-inhibitor.

## Discussion

Due to high morbidity and mortality, the CoA should be diagnosed and corrected early in life. The common complications of un-treated CoA in adults include hypertension, premature coronary artery disease, sudden cardiac death, heart failure, stroke, endocarditis, rupture, dissection, aneurysm and cardiovascular disease [4–8]. However, due to rarity in adults, benefits of early repair are unclear. Therapeutic goals in adult patients include symptomatic improvement, control of hypertension and an intervention which is suitable for individual patient.

So far, there is no consensus on the indications of surgical and percutaneous interventions in older patients [10–13]. Historically, the mortality rate in unrepaired CoA is 90 % by 50–years of age, with a mean age of 35 years [7–9].

In view of the rapid advance in the imaging and interventional technologies, the number of such case reports may increase in future. Therefore, it is pertinent to discuss similar cases for a better understanding and management. Traditional surgical resection, patch repair and interposition graft placement techniques are well known for an uncomplicated juvenile CoA [8–10]. However, due to high risks involved with invasive procedures in adult population, a minimal invasive approach such as percutaneous intervention is a preferred therapeutic approach in recent times. The tissue characteristics, chronic nature of the disease, associated co-morbid conditions and collateral circulation makes adult population a high–risk group for all interventions especially the surgical ones [6, 10].

The initial percutaneous approach in the treatment of the coarctation of the aorta was balloon angioplasty but endovascular stent placement is gaining wider acceptance recently. The incidence of early and late aneurysm after balloon angioplasty has been reported to be 5–11 % in different series [6, 12, 13]. This has led to the development of balloon-expandable stents and presently there are covered stents available in the market as a further advancement. These stents provide support to the vessel wall, prevent aneurysm formation and amenable to re-dilatation [6].

Fluoroscopy guided catheter perforation of the atretic membrane in the aortic lumen lumen through a combined antegrade and retrograde ‘kissing’ technique via axillary or femoral trans-septal approaches have been described for stenting [3–5]. We could incidentally managed a gentle perforation of the atretic membrane using the stiff end of the Terumo wire passed through the distal end of the involved aorta, thereby, avoided a second vascular access. The presence of atretic coarctation makes transcatheter interventions, a high–risk procedure due to anticipated inadvertent perforation or failure of the procedure. Therefore, it is prudent to have a surgical backup available.

In our patient, we have used the covered Cheatham-Platinum stent due to expected benefit in terms of a better long-term result by reducing the possibility of recurrence or re-intervention [6]. The maximum balloon diameter is usually chosen based on the diameter of either the distal aortic arch or the transverse arch with a maximum oversize of approximately two mm greater. The length of the stent is calculated as per the distance between the origins of the left subclavian artery to a point approximately 15 mm beyond the site of coartation distally.

A review of the recently published literature has shown the short and intermediate-term benefits of transcatheter stenting techniques (Table 1). However, the possibility of re-coarctation and need for re-intervention remains higher [6–13]. The actuarial freedom from the re-intervention at 60-months after intervention using stents is approximately 95 % [11]. However, overall short-term complication rate for endovascular interventions are 32.1 % for balloon angioplasty and 8.3 % for stenting [12]. Similarly, the overall long-term complication rates for balloon interventions were 43.8 % as compared to 12.5 % in stent patients [12].

**Table 1:** Follow up data of patients with transcatheter intervention for coarctation of the aorta in adults

Author	Year	Number of patients	Age range (years)	Mean gradient across the coarctation after the procedure (mm Hg)	Mean increase in diameter of the involved segment (mm)	Patients with improvement in hypertension after the procedure (%)	Follow up period (months)
Tzifa 'et–al'	2006	30 (covered stents)	28 ± 17.5	4 ± 4	17.1 ± 3.1	43	11
Shennib 'et–al'	2010	22 (13 balloon and stents, 9 endoluminal grafts)	40 + 18	4 + 7	19 + 4	100	31 + 15.6
Forbes 'et–al'	2011	61 (ballon angioplasty)	0.4–42.5	≤10 in 55 % patients	Coarctation: Descending aorta diameter ratio ≥ 0.6 in 93 % patients	16 % patients on anti hypertensive medications	18–60
				≤15 in 82 % patients			
Forbes 'et–al'	2011	217 (stents)	2.2–74.3	≤10 in 75 % patients	Coarctation: Descending aorta diameter ratio ≥ 0.6 in 89 % patients	31 % patients on anti hypertensive medications	18–60
				≤15 85 % patients			

The common long-term complications of transcatheter interventions are aortic wall injury, dissection, aneurysm, re-coarctation or stent fracture [11–13]. The recurrence of coarctation is attributed to the severity of the disease, fibrotic changes, cystic medial necrosis, intimal tear, thrombosis and graft-infolding in the involved redundant aortic segment due to oversizing during initial balloon dilatation or stent placement. The covered stents may minimize such complications due to the fact that not only it stabilize the aortic wall but can be re-dilated, as in our patient.

The covered stents are valuable and recent addition in the management strategies; therefore, more long-term data is required to establish their role.

## Conclusion

Transcatheter stent dilatation is a safe and acceptable alternative to surgical repair or balloon angioplasty in complex atretic CoA. Early and intermediate outcomes associated with transcatheter interventions including covered stents appear to be promising^.^ However, there is a need to explore new frontiers in the management of adult coarctation of aorta in order to refine the existing techniques or prevent complications by modifying the factors influencing short or long-term outcomes.

## Consent

Written informed consent was obtained from the patient for publication of this case report and any accompanying images. A copy of the written consent is available for review by the Editor-in-Chief of this journal.

## Abbreviations

CoA: coarctation of aorta
NYHA: New York heart association
ACE: angiotensin converting enzyme
AO: ascending aorta
DO: descending aorta
PTFE: polytetrafluoroethylene
CT: computerized tomography

## References

[1] Grech V (1999). Diagnostic and surgical trends, and epidemiology of coarctation of the aorta in a population-based study. Int J Cardiol.

[2] Campbell M (1970). Natural history of coarctation of the aorta. Br Heart J.

[3] Musso TM, Slack MC, Nowlen TT (2008). Balloon angioplasty with stenting to correct a functionally interrupted aorta: A case report with three-year follow-up. Catheter Cardiovasc Interv.

[4] Sunumlari O (2012). Transcatheter antegrade perforation and covered stent implantation to subatretic coarctation. Anadolu Kardiyol Derg.

[5] Sreeram N, DeGiovanni J (2005). Stent implantation for coarctation facilitated by the antegrade trans-septal approach. Images Paediatr Cardiol.

[6] Tzifa A, Ewert P, Rajszys GB, Peters B, Bjoern P, Zubrzycka M (2006). Covered Cheatham-Platinum stents for aortic coarctation. early and intermediate-term results. J Am Coll Cardiol.

[7] Ohlow MA, Lauer B (2013). Coarctation of aorta with complete occlusion. J Geriatric Cardiol.

[8] Cohen M, Fuster V, Steele PM, Driscoll D, McGoon DC (1989). Coarctation of aorta. Long term follow up and prediction of outcome after surgical correction. Circulation.

[9] Forbes TJ, Gowda ST (2014). Intravascular stent therapy for coarctation of the aorta. Methodist Debakey Cardiovasc J.

[10] AlKhaldi A, Alhabshan F, Tamimi O, Jha N (2008). Repair of aortic arch atresia with diffuse hypoplasia of the descending thoracic aorta. European J Cardiothorac Surg.

[11] Shennib H, Lopez JR, Ramaiah V, Wheatley G, Kpodonu J, Williams J (2010). Endovascular management of adult coarctaion and its complications: intermediate results in a cohort of 22 patients. European J Cardiothorac Surg.

[12] Forbes TJ, Kim DW, Du W, Turner DR, Holzer R, Amin Z (2011). Comparison of surgical, stent, and balloon angioplasty treatment of native coarctation of the aorta. an observational study by the CCISC (Congenital Cardiovascular Interventional Study Consortium). J Am Coll Cardiol.

[13] Godart F (2011). Intravascular stenting for the treatment of coarctation of the aorta in adolescent and adult patients. Archives of Cardiovascular Disease..

